# Organic vs Stimulant-Induced Psychosis in the Peripartum Period

**DOI:** 10.7759/cureus.10718

**Published:** 2020-09-29

**Authors:** Michelle Zaydlin, Christina Tamargo

**Affiliations:** 1 Psychiatry, University of Miami Miller School of Medicine, Jackson Memorial Hospital, Miami, USA; 2 Medicine, University of Miami Miller School of Medicine, Jackson Memorial Hospital, Miami, USA

**Keywords:** psychosis, methamphetamine use, pregnancy, case report, psychiatry, psychiatry & mental health, peripartum psychosis, substance-induced disorders

## Abstract

This is the case of a 30-year-old pregnant female who presented to the medical emergency department with signs and symptoms consistent with a psychotic episode. At the time of presentation, the patient was noted to have paranoia and delusions focused on her current pregnancy. On further evaluation in the emergency department, the patient’s urine toxicology was found to be positive for methamphetamines. Following medical clearance, the patient was admitted for acute inpatient psychiatric stabilization. During hospitalization, her psychosis rapidly resolved with only the use of two emergency treatment injections containing antipsychotic medication. This case presents an interesting differential diagnosis between a brief psychotic disorder with peripartum onset and a substance-induced psychosis, and allows for further discussion in the differentiation and clinical treatment of these diagnoses.

## Introduction

According to the Diagnostic and Statistical Manual of Mental Disorders, 5th Edition (DSM-5, 2013), disorders with abnormalities including hallucinations, disorganized speech, disorganized, abnormal or catatonic behavior, and negative symptoms such as flat affect, poverty of speech, and anhedonia are classified as schizophrenia spectrum and other psychotic disorders. Subtypes exist to better classify each disorder in terms of symptom content, episode frequency, time of onset, and other factors [1], and allow for improved communication between providers to improve treatment strategies. Psychotic disorders can occur throughout the lifespan, normally beginning in early adulthood, and encompass a wide range of clinical diagnoses. Psychotic disorders that are triggered during pregnancy (peripartum) or after pregnancy (postpartum), although rare, are considered psychiatric emergencies due to risk of harm to both the mother and the child [2].

Like many psychiatric disorders, a combination of biopsychosocial factors contributes to peripartum disorders. It is believed that these disorders may be associated with abnormalities in maternal neurotransmitters, alterations in estrogen levels due to pregnancy, hypothalamic-pituitary-adrenal axis dysfunction, and thyroid dysfunction, along with genetic predisposition [3]. Still, the exact pathophysiology and risk factors for peripartum disorders, in particular peripartum psychosis, remain unknown. However, there are other conditions that when manifested during pregnancy may need to be considered as differential diagnoses. In particular, substance use can lead to a substance-induced psychotic disorder (SIPD), also presenting with delusions and/or hallucinations, which may complicate the diagnosis of brief psychotic disorder with peripartum onset [1,4].

In addition to organic psychiatric disorders, acute intoxication and withdrawal from substances can also present with psychotic symptoms. The treatment of psychotic symptoms often requires careful analysis and differentiation between primary psychotic disorders and substance-induced psychoses, the latter of which tend to resolve once the substance is out of the system. 

## Case presentation

This is a case of a pregnant, G5P3A2, 30-year-old female with no clear past psychiatric history who presented to the medical emergency room requesting removal of her unborn child, stating it “was a devil snake inside of her, she can feel it moving and wants it out.” The patient presented alone and voluntarily to the medical emergency department (ED) where she was noted to be acutely psychotic, disorganized, noncooperative, and with delusions focused on her unborn child. Per chart review, family history was noncontributory. The patient was hemodynamically stable, and physical exam was within normal limits. Laboratory examination was grossly unremarkable except for urine toxicology, which was positive for amphetamines. At this time, the patient was unable to elaborate regarding substance use given her clinical condition and chart review did not reveal a history of amphetamine use. In the ED, ultrasound was completed that demonstrated a healthy 27-week fetus. During evaluation by the internist and obstetrician, the patient remained verbally aggressive and combative, requiring emergency medication due to risk of harm to self and others. Haloperidol 5 mg, lorazepam 1 mg, and diphenhydramine 50 mg were administered via intravenous (IV) push. The patient was placed on an involuntary hold for psychiatric evaluation and remained with a 1:1 sitter for safety until medical workup was completed and the patient could be transferred to the behavioral health emergency department. Of note, based on review of the patient’s electronic medical record, she had last been evaluated approximately five weeks prior for routine obstetric care, at which time she was calm, cooperative, and noted without changes in mood or perceptual disturbances. 

Following medical clearance, the patient was transferred to the behavioral health ED. Upon presentation, the patient was noted to be somnolent and unable to engage in meaningful interview secondary to administration of medication in the medical emergency department. Upon further psychiatric evaluation, the patient continued with the above-mentioned psychotic symptoms and, due to risk of harm to self and others, she admitted involuntarily to acute inpatient psychiatry. 

On the first day of admission, the patient was noted to be isolative with limited interaction with staff and peers on the inpatient unit. On initial interview with the primary inpatient psychiatric treatment team, the patient was alert and oriented to self and time but disoriented to place. She was irritable, disorganized, and noted to be a poor historian. The patient continued to perseverate on the need for removal of her unborn fetus, stating that “the snake was biting her and causing a current to run through her body.” The patient endorsed command auditory hallucinations to “remove the snake”; she was distractible throughout the interview and appeared to be responding to internal stimuli. She also endorsed visual hallucinations of a male figure whom she was “unable to catch.” She demonstrated poor insight into her condition and continued to represent a risk to self and others; psychiatric hospitalization was thus continued under involuntary status. 

Later that night, the patient again became acutely agitated, threatening staff on the unit and attempting to elope. Despite multiple attempts at redirection and verbal de-escalation, the patient continued to present acute risk of harm to self and others, requiring emergency medication. Haloperidol 5 mg and diphenhydramine 50 mg, the same medications that had been administered in the emergency department, were administered intramuscularly (IM). Following medication administration, the patient was able to sleep through the night with no further issues. 

The following morning, the patient’s demeanor had changed. She was notably less irritable and was superficially cooperative on interview with the primary psychiatric treatment team. The patient denied stating she thought the baby was a snake, reporting instead that it “just felt like a snake,” However, her desire to gain discharge was apparent, as she asked questions such as, “What else do I need to say to leave?” Shortly after the interview, the patient was observed attempting to elope from the inpatient unit while verbally and physically threatening unit staff. Another emergency treatment dose of haloperidol 5 mg and diphenhydramine 50 mg was administered IM. Given the continued episodes of unsafe behavior requiring emergency medication and the patient’s continued poor insight and judgment, she continued to present a risk of harm to herself and others, requiring further hospitalization. 

On interview the following day, the patient’s psychosis appeared to have improved significantly. She was calm, cooperative, with linear and logical thinking. She no longer fixated on being pregnant with a snake and was able to recognize and verbalize that she was pregnant with a healthy fetus. Upon further discussion regarding her positive urine toxicology from admission, the patient reported that she had abstained from substances of abuse for “many months,” but relapsed the night prior to admission. She attributed her presentation of bizarre behavior and acute psychosis to her substance use and stated that her symptoms had now resolved. The patient was goal-directed and future-thinking, reporting wanting to be with her family for the remainder of pregnancy and at time of childbirth. At that time she was counseled regarding substance use, particularly during pregnancy, and expressed understanding. She denied other substance use history during pregnancy and declined to elaborate on amphetamine use prior to presentation, stating it was a "one time incident." She did not require any further emergency treatment throughout her admission and continued to deny thoughts of harm to self or others, including the baby; she also stated she planned to remain abstinent from substances of abuse after discharge. The patient was discharged after a six-night admission with follow-up appointments with an obstetrician and psychiatrist, as well as referrals and resources for substance abuse. 

## Discussion

While pregnancy is a positive experience for many mothers, for some it can be dangerous. In addition to the physical challenges that pregnancy places on a woman’s body, it also increases a woman’s susceptibility to a variety of psychiatric conditions. These may include postpartum blues, peri- and postpartum depression, and brief psychotic disorder with peripartum onset [5]. The last of these encompasses both pre- and postpartum psychosis and has a recorded incidence rate of less than 0.5% with occurrence increased to 28% of women with a history of psychosis [6-8]; however, it is increasingly rare in women with no previous psychiatric history, such as this patient [5]. In fact, many consider brief psychotic disorder with peripartum onset to be a manifestation of an underlying psychiatric disorder, most commonly bipolar or schizoaffective disorder, and less commonly schizophrenia [9,10].

Additionally, prepartum psychosis is much less common, and much less studied, than postpartum psychosis [5]. In 1990, Brockington et al. reported four cases of prepartum psychosis and suggested that they were “evidence for the prepartum onset of postpartum psychosis,” suggesting perhaps that prepartum psychosis was rare, if not nonexistent, on its own accord [7]. Moreover, most cases of brief psychotic disorder with peripartum onset are within four weeks of delivery. While initially this patient draws a clinical picture of prepartum psychosis, her lack of previous psychiatric history, the timing of her psychotic episode, and its rapid resolution with limited psychotropic medication bring this into question. 

The other diagnosis that one must consider in any psychotic patient, pregnant or not, with recent history of drug use is SIPD. The DSM-5 definition of SIPD requires the presence of delusions and/or hallucinations while or soon after an individual uses a substance capable of producing such psychotic symptoms [1]. Importantly, a diagnosis of SIPD requires that these symptoms are not explained by another psychiatric disorder. In the case of this patient, the differentiation is unclear as to whether is this a case of SIPD that resolved with abstinence from amphetamines or is it a unique manifestation of brief psychotic disorder with peripartum onset that resolved with antipsychotic therapy.

Many studies have demonstrated the ability of amphetamines to trigger acute psychosis in a variety of patient cohorts, from healthy individuals to regular users of amphetamines [4,11,12]. Amphetamine binges have been shown to often end in psychosis, and symptoms of this psychosis range from delusions to anxiety to auditory and visual hallucinations, among many other manifestations [11-13]. While many of these symptoms are identical to those of schizophrenia, acute amphetamine-induced psychosis has a faster recovery and typically improves or resolves with abstinence [4,14-18].

Additionally, much research has demonstrated that patients with primary psychotic disorders are at greater risk for substance abuse, including amphetamines [19]. Even further, Japanese researchers suggest that amphetamine-induced psychosis could last for several years, with relapses triggered by stressful situations [20]. 

This makes the patient’s diagnosis all the more challenging. This is a patient who may have had an underlying psychiatric illness that predisposed her to psychosis in the setting of substance abuse, regardless of her current pregnancy. She also may have had undisclosed amphetamine-induced psychosis in the past that resurfaced during pregnancy, an obvious physiological stressor. Other diagnostic possibilities in this case include a peripartum psychotic episode that rapidly resolved with administration of three doses of psychotropic medication or a combination of multiple biopsychosocial factors leading to an acute psychotic episode. This case presents an opportunity for interesting discussions regarding overlapping psychiatric presentations and the role of substances and pregnancy in the manifestation of psychiatric disorders.

## Conclusions

The presentation of psychosis in the peripartum period presents an interesting and often challenging diagnostic picture. Brief psychotic disorder with peripartum onset may be an independent disorder, but rarely presents in patients with no past psychiatric history. Rather, most patients presenting with this condition have underlying psychotic disorders, such as bipolar disorder, schizoaffective disorder, or schizophrenia. The picture is further complicated when the mother is using substances of abuse, which themselves can lead to a substance-induced psychosis. Ultimately, the goal in any patient presenting with psychotic symptoms is to ensure the safety of the patient and those around them, such as the fetus in pregnant patients, regardless of the final diagnosis.

There is an extreme lack of studies and information on prepartum psychosis, substance use in pregnancy, and psychiatric disorders in pregnancy in general. This makes an already complex differential diagnosis even more challenging. Future research in psychotic disorders during pregnancy, especially those complicated by substance use, is needed and will continue to improve patient care and outcomes.
